# Trends in Heart Rate and Heart Rate Variability During Pregnancy and the 3-Month Postpartum Period: Continuous Monitoring in a Free-living Context

**DOI:** 10.2196/33458

**Published:** 2022-06-03

**Authors:** Fatemeh Sarhaddi, Iman Azimi, Anna Axelin, Hannakaisa Niela-Vilen, Pasi Liljeberg, Amir M Rahmani

**Affiliations:** 1 Department of Computing University of Turku Turku Finland; 2 Department of Nursing Science University of Turku Turku Finland; 3 Department of Obstetrics and Gynaecology Turku University Hospital Turku Finland; 4 Faculty of Medicine University of Turku Turku Finland; 5 Department of Computer Science University of California, Irvine Irvine, CA United States; 6 School of Nursing University of California, Irvine Irvine, CA United States; 7 Institute for Future Health University of California, Irvine Irvine, CA United States

**Keywords:** heart rate, heart rate variability, pregnancy, postpartum, continuous monitoring, PPG, mobile phone

## Abstract

**Background:**

Heart rate variability (HRV) is a noninvasive method that reflects the regulation of the autonomic nervous system. Altered HRV is associated with adverse mental or physical health complications. The autonomic nervous system also has a central role in physiological adaption during pregnancy, causing normal changes in HRV.

**Objective:**

The aim of this study was to assess trends in heart rate (HR) and HRV parameters as a noninvasive method for remote maternal health monitoring during pregnancy and 3-month postpartum period.

**Methods:**

A total of 58 pregnant women were monitored using an Internet of Things–based remote monitoring system during pregnancy and 3-month postpartum period. Pregnant women were asked to continuously wear Gear Sport smartwatch to monitor their HR and HRV extracted from photoplethysmogram (PPG) signals. In addition, a cross-platform mobile app was used to collect background and delivery-related information. We analyzed PPG signals collected during the night and discarded unreliable signals by applying a PPG quality assessment method to the collected signals. HR, HRV, and normalized HRV parameters were extracted from reliable signals. The normalization removed the effect of HR changes on HRV trends. Finally, we used hierarchical linear mixed models to analyze the trends of HR, HRV, and normalized HRV parameters.

**Results:**

HR increased significantly during the second trimester (*P*<.001) and decreased significantly during the third trimester (*P*=.006). Time-domain HRV parameters, average normal interbeat intervals (IBIs; average normal IBIs [AVNN]), SD of normal IBIs (SDNN), root mean square of the successive difference of normal IBIs (RMSSD), normalized SDNN, and normalized RMSSD decreased significantly during the second trimester (*P*<.001). Then, AVNN, SDNN, RMSSD, and normalized SDNN increased significantly during the third trimester (with *P*=.002, *P*<.001, *P*<.001, and *P*<.001, respectively). Some of the frequency-domain parameters, low-frequency power (LF), high-frequency power (HF), and normalized HF, decreased significantly during the second trimester (with *P*<.001, *P*<.001, and *P*=.003, respectively), and HF increased significantly during the third trimester (*P*=.007). In the postpartum period, normalized RMSSD decreased (*P*=.01), and the LF to HF ratio (LF/HF) increased significantly (*P*=.004).

**Conclusions:**

Our study indicates the physiological changes during pregnancy and the postpartum period. We showed that HR increased and HRV parameters decreased as pregnancy proceeded, and the values returned to normal after delivery. Moreover, our results show that HR started to decrease, whereas time-domain HRV parameters and HF started to increase during the third trimester. The results also indicated that age was significantly associated with HRV parameters during pregnancy and postpartum period, whereas education level was associated with HRV parameters during the third trimester. In addition, our results demonstrate the possibility of continuous HRV monitoring in everyday life settings.

## Introduction

### Background

Heart rate variability (HRV) reflects alterations in the regulation of the autonomic nervous system. Substantial changes in autonomic nervous system, by implication in HRV, occur during pregnancy. Such physiological changes help to ensure the healthy development of the fetus [1]. Heart rate (HR) increases during pregnancy [2], whereas HRV parameters decrease; however, the values usually return to normal within a few months of the postpartum period [3-5].

In addition to physiological causes, changes in HRV during pregnancy may also reflect other issues; for example, certain physical or mental complications. Previous studies have shown that HRV during pregnancy may indicate hypertensive disorders [6,7] or pre-eclampsia [8,9]. Pregnant women with gestational hypertension have higher low-frequency power (LF) to high-frequency power (HF) ratio (LF/HF) in early pregnancy than those with normal pregnancies [6]. Regarding pre-eclampsia, women have lower HF than those with normal pregnancies, resulting in an increase in LF/HF in pre-eclamptic pregnancies [8,9]. Furthermore, HRV may reflect the state of mental health in pregnant women; the effects of depression [10] and anxiety [11,12] on HRV parameters during pregnancy have been studied. Pregnant women with depression have low 24-hour time-domain parameters [10], and anxiety during pregnancy has been shown to decrease HF and very low–frequency power [11]. HRV parameters may also illustrate the level of stress experienced by pregnant women [13,14]. Induced stress has been shown to decrease HF in pregnant women. Symptoms of anxiety may further be associated with stress, as pregnant women with anxiety had dampened stress reactivity [12]. In addition, the decrease in root mean square of the successive difference of normal interbeat intervals (IBIs; RMSSD) and HF was significantly less in mindful pregnant women who have better resources to cope with stress during pregnancy [15]. All pregnancy-related complications are important to be detected early in maternity care, to enable appropriate interventions to secure the health of the pregnant woman and her fetus. However, interpretation of HRV is demanding owing to the complexity of the human cardiac system; changes in and the behavior of HRV varies across individuals.

HRV parameters are usually measured using electrocardiogram (ECG) or photoplethysmogram (PPG). Electrocardiography is a noninvasive method for monitoring the electrical activity of the cardiovascular system using electrodes attached to the skin. It is the gold standard for monitoring HR and HRV parameters, but cannot be used for long-term monitoring. In contrast, photoplethysmography is an optical method for monitoring heart activity and is more convenient for use in home and free-life settings. It is an easy-to-implement method that is used in many clinical and commercial wearable devices. Therefore, it is increasingly used in remote health monitoring systems.

Most studies have investigated changes in HRV in an episodic manner, using 1-time ECG recording of pregnant women at different gestational weeks or during labor [16-20]. In addition, in most longitudinal studies, 10 to 30 minutes of ECG were recorded from pregnant women once per trimester or monthly during pregnancy [7,21-24] and postpartum period [4]. The recordings were performed while the women were resting in a predefined position (usually supine position) in a laboratory setting. Stein et al [1] conducted 24-hour HRV recordings with pregnant women 4 times during pregnancy and once before pregnancy. Continuous measurements of HRV during pregnancy and early postpartum period may provide new and valuable information about the HRV patterns.

Although existing studies have characterized changes in HRV during pregnancy and the postpartum period, they have been limited to short-time recordings of ECG signals a few times during pregnancy. Some of these studies compared pregnant women at different gestational weeks with nonpregnant women to identify HRV trends. However, comparing HRV from different individuals can be inaccurate because HRV is unique for each person and is dependent on various parameters such as age and sex among many other factors [25]. In addition, other studies have collected few ECG recordings from the same individuals. Thus, owing to the limited number of measurements, the results cannot reliably capture the changes. Moreover, only Stein et al [1] collected data in home-based settings, whereas all the other studies used laboratory settings to collect HRV parameters.

### Objectives

In this paper, we aimed to analyze the nighttime HRV trends during pregnancy and postpartum period. To the best of our knowledge, this study is the first to collect continuous PPG signals from pregnant women, in everyday settings over a long period. We used an Internet of Things (IoT)-based system to collect PPG signals from 58 women, several times a day during pregnancy and the first 3-month postpartum period. The continuous monitoring of HRV parameters enabled us to accurately detect HRV trends regarding in-person and between-person differences. Moreover, we analyzed the trends of normalized HRV parameters. The normalization was performed based on average HR to remove the effect of HR changes on HRV parameters. In addition, we added age, BMI, and education level to our analysis as controlling factors and analyzed their effects on HRV trends. In summary, the contributions of this study were as follows:

1. Continuous monitoring of HRV in pregnant women, in everyday settings using a customized, remote, IoT-based monitoring system.
2. Analyzing HRV trends during pregnancy and postpartum period during the night.
3. Analyzing normalized HRV trends during pregnancy and postpartum period during the night.

## Methods

### Study Design

HRV parameter trends during pregnancy and postpartum period were investigated in a longitudinal study using an IoT-based system. The system used a smartwatch to remotely collect HRV parameters and a cross-platform mobile app to collect background and delivery-related information. The collected data were transferred to the cloud server for further analysis. The use of such a home-based system during pregnancy and postpartum period was evaluated in a previous study [26]. The findings of this pilot study indicated the feasibility of the study, robustness of the system, and reliability of the collected HRV parameters.

### Participants and Recruitment

Women with singleton pregnancies who were at 12 to 15 weeks of gestation were recruited from southwest Finland. Women with both high-risk and low-risk pregnancies were recruited. Women with high-risk pregnancies were required to have a history of preterm birth (22-36 weeks of gestation) or late miscarriage (12-22 weeks of gestation). Women with low-risk pregnancies were required to have a history of full-term uncomplicated pregnancy and no pregnancy loss. All eligible participants had to be aged ≥18 years, understand Finnish, and have a smartphone running Android or iOS. The recruitment goal for each group (high risk and low risk) was 30 participants, for a targeted total of 60 participants.

Recruitment was performed via advertisements on social media and in maternity clinics from January 2019 to March 2021. The researcher scheduled face-to-face meetings with eligible pregnant women. During the meetings, the pregnant women were informed about the objective of the study. After the participants provided written informed consent, they were provided with a smartwatch and instructions. Moreover, our customized cross-platform mobile app was installed on their smartphone. Participants were asked to wear the smartwatch continuously during pregnancy and for 3 months after delivery. A total of 62 women were recruited (n=32, 52% in the high-risk group and n=30, 48% in the low-risk group), but 13% (4/32) of the women in the high-risk group withdrew from the study. Finally, all participants in both the high-risk and low-risk pregnancy groups were combined into 1 group for the analyses because there were no significant differences in their HRV trends. Table 1 shows the participants’ background information.

**Table 1:** Participants’ background information (n=58).

Parameters			Values
Age (years), mean (SD)			31.9 (4.9)
BMI (kg/m^2^), mean (SD)			25.98 (5.96)
**Marital status, n (%)**			
	Married or cohabitation	57 (98)	
	Other	1 (2)	
**Work status, n (%)**			
	Working	44 (76)	
	Student	7 (12)	
	Unemployed	1 (2)	
	Other	6 (10)	
**Education, n (%)**			
	High school	24 (41)	
	College	18 (31)	
	University	16 (28)	
**Pregnancy planned, n (%)**			
	Yes	53 (91)	
	No	5 (9)	
Duration of pregnancy at recruitment (week+day), mean (SD)			14+3 (1+4)
Duration of pregnancy at birth (week+day), mean (SD)			36+4 (9+6)
**Mode of delivery, n (%)**			
	Vaginal	48 (83)	
	Cesarean	10 (17)	
Infant birth weight (g), mean (SD)			3532.7 (561.2)

### Data Collection

Data collection was performed using the Samsung Gear Sport smartwatch and a cross-platform mobile app. The lightweight smartwatch was chosen based on its onboard sensors, battery life, configurability, internal memory, and processing unit. Moreover, the smartwatch provided access to raw PPG signals and enabled continuous data collection. The watch runs the Tizen operating system, which is open source. The open-source platform enabled us to develop customized data collection applications for the watch. We used the smartwatch to collect 12 minutes of PPG signals every 2 hours at a sampling frequency of 20 Hz. The setup was selected to enable battery life of 2 to 3 days after each full recharge [26]. The collected data were stored on the internal storage of the smartwatch. We also developed an application for the smartwatch to send the data manually through the Wi-Fi connection to our cloud server. We asked the participants to upload their data regularly. The internal storage was sufficient to store the collected data for 2 months. However, we sent notifications to the participants if they did not upload the data for 2 weeks. In addition, a cross-platform mobile app collected background information about pregnancy and infant-related data after delivery.

We collected PPG signals for extracting HRV parameters. Nighttime PPG data were used in this study to extract the HRV trends during pregnancy and postpartum period. Daytime PPG data were discarded as participants were involved in various activities and environments during the day, making PPG signals unreliable owing to movement artifacts and environmental noises.

### Data Analysis

#### Overview

**Figure 1:**
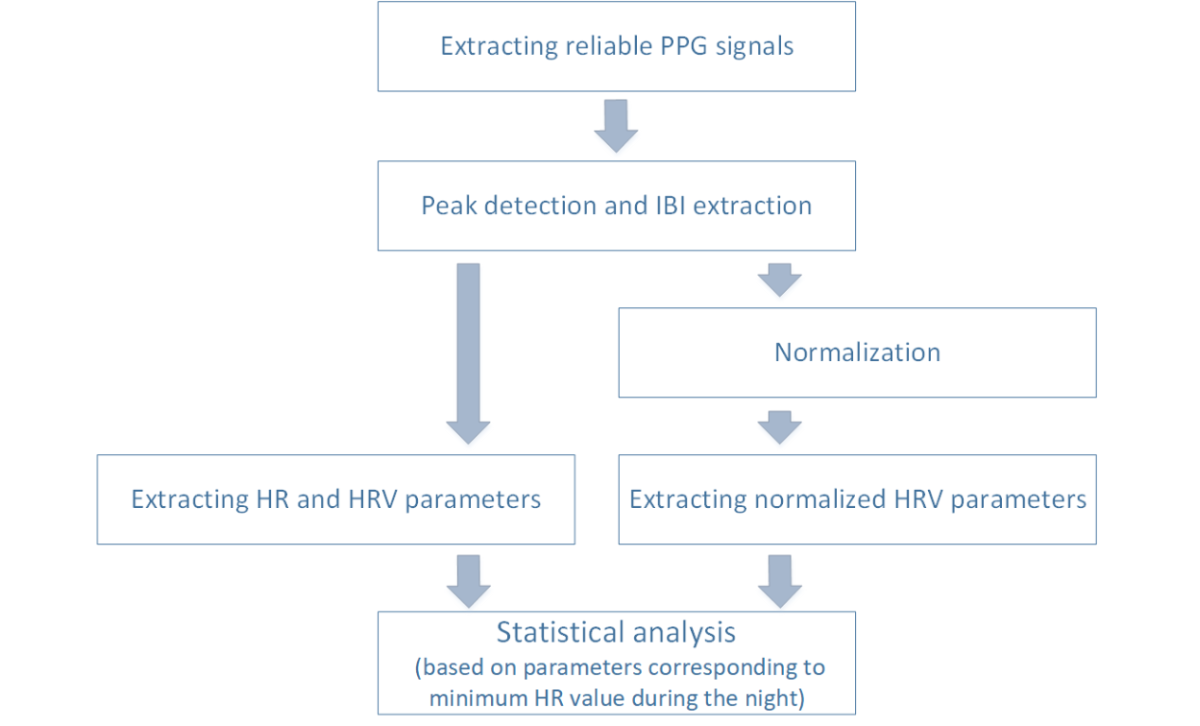
Data analysis pipeline. HR: heart rate; HRV: HR variability; IBI: interbeat interval; PPG: photoplethysmogram.

We analyzed the collected data on the cloud server. Data analysis included several steps, as shown in Figure 1. First, we identified and extracted reliable PPG signals. Then, a peak detection method was used to extract the peaks and IBIs. In the next step, we normalized the reliable signals to reduce the effect of HR changes on HRV parameters (refer to the *Parameter Normalization* section). We used reliable signals and normalized signals to extract reliable HR and HRV parameters. Then, we leveraged the HRV parameters during the nighttime, when resting HR has the lowest value and artifacts would be minimum to analyze HRV trends during pregnancy and the postpartum period. Finally, we used hierarchical linear mixed (HLM) models to analyze the trends of HRV parameters during pregnancy and the postpartum period.

#### Extracting Reliable Signals

PPG is a noninvasive optical method for extracting HR and HRV parameters. This method is easy to implement, and many wearables include PPG sensors. However, this method is susceptible to environmental noise and motion artifacts. Such noise can affect the quality of the signal and the accuracy of the analysis [27,28]. Therefore, unreliable signals must be detected and discarded. Reliable signals are expected to have similar waveforms, whereas unreliable signals have diverse waveforms as they are affected by different motion artifacts and environmental noises [29]. First, we extracted several morphological features from the signals and heart cycles. Then, we chose skewness, kurtosis, approximate entropy, Shannon entropy, and spectral entropy based on the scoring value for clustering [29]. We trained a support vector machine classifier using these features to distinguish between the reliable and unreliable PPG signals. Using this classifier, we discarded unreliable signals and used reliable signals in our analysis.

#### Peak Detection and IBI Extraction

We used a bandpass filter with cutoff frequencies of 0.7 Hz and 3.5 Hz to enhance PPG signals by filtering noises that are not in human HR ranges. Then, we used the peak detection method based on the moving average, as described in [30], to find the peaks that correspond to heartbeats. The method is enabled by an adaptive threshold, which considers the variations in the morphology and amplitude of PPG signals [31]. Then, the detected peaks were used to extract IBI, which is the interval between 2 consecutive peaks. In the error detection phase, IBIs that deviated >30% from the mean IBIs of the segments (5 minutes of signals) were removed from the IBI lists. We leveraged the HeartPy library in Python for this analysis [31].

#### Parameter Normalization

Studies suggest that HRV parameters are significantly associated with average HR [32]. Therefore, changes in HRV parameters result from changes in HR or HR variation [32]. In addition, several studies have found that HR increases during pregnancy [1,2]. We normalized the HRV parameters based on HR to cancel the inevitable effect of HR changes on HRV parameters. Moreover, normalization is required to compare the HRV parameters of different people, because each person’s HR and resting HR are unique. Normalization was performed by dividing the IBIs by the corresponding average IBI values [33].

### HR and HRV Extraction

We used the detected peaks to extract HR, the number of peaks (heartbeats) per minute. HRV parameters were obtained by extracting the variation in IBIs and normalized IBIs in the PPG signals. We used short-term HRV analysis, which requires 5-minute recordings of reliable PPG signals [25,34]. We leveraged the IBIs in each 5-minute window of reliable PPG signals to extract time-domain HRV parameters, including average of normal IBIs (AVNN), RMSSD, and SD of normal IBIs (SDNN), and frequency-domain parameters, including LF (power in low-frequency range), HF (power in high-frequency range), and LF/HF (LF to HF ratio). These parameters can be reliably extracted at a sampling frequency of 20 Hz [35].

The time-domain HRV parameters show the variation in IBIs during the monitoring period. The SDNN in the 5-minute resting measurements mainly shows the variation in parasympathetically-mediated respiratory sinus arrhythmia. RMSSD reflects the variation in successive normal IBIs. Moreover, RMSSD is the most commonly used HRV parameter for investigating vagal changes [25].

The frequency-domain HRV parameters decompose the HRV to different frequency ranges. LF can be produced by both parasympathetic and sympathetic systems. HF reflects the parasympathetic nervous system and is correlated with RMSSD [25]. Moreover, LF/HF shows the ratio of LF to HF.

We also computed the corresponding normalized parameters discussed in the *Parameter Normalization* section. The normalization resulted in normalized SDNN (nSDNN), normalized RMSSD (nRMSSD), normalized LF (nLF), normalized HF (nHF), and nLF/nHF. The HRV parameters used in this study and their definitions are presented in Table 2.

**Table 2:** Heart rate variability parameters.

Parameter and types			Unit		Description
**Time-domain**					
	AVNN	ms		Average of normal IBIs^a^	
	SDNN	ms		SD of normal IBIs	
	nSDNN	ms		SD of normalized IBIs	
	RMSSD	ms		Square root of the mean of the sum of the squares of differences between adjacent normal IBIs	
	nRMSSD	ms		Square root of the mean of the sum of the squares of differences between adjacent normalized IBIs	
**Frequency-domain**					
	LF	ms^2^		Power in low-frequency range (0.04-0.15 Hz)	
	nLF	ms^2^		Power in low-frequency range (0.04-0.15 Hz) in normalized IBIs	
	HF	ms^2^		Power in high-frequency range (0.15-0.4 Hz)	
	nHF	ms^2^		Power in high-frequency range (0.15-0.4 Hz) in normalized IBIs	
	LF/HF	N/A^b^		Ratio of LF to HF	
	nLF/nHF	N/A		Ratio of nLF to nHF	

^a^IBI: interbeat interval.

^b^N/A: not applicable.

### Statistical Analysis

We used HLM models [36,37] to analyze the trends in the HRV parameter. The HLM method considers within-person and between-person changes. The HLM model supports multilevel statistical analysis when we have repeated measurements that are not independent and can correctly model correlated errors [36]. This model assumes a linear relationship between dependent and independent variables. It also enables hierarchical analysis and comparison of continuous dependent variables during different time frames (eg, before-after studies) [36].

We used the HLM models to evaluate the changes in HRV parameters during monitoring. We investigated trends in the second trimester (16-28 weeks of gestation), third trimester (29-40 weeks of gestation), and postpartum period (12 weeks after delivery). In the HLM models, HRV parameters were treated as dependent variables and time (days) was the independent variable. Therefore, the HLM model investigated HRV trends in the desired period while considering the dependency of the measurements from individual participants. We also used background parameters including age, BMI, and education level as controlling factors and analyzed their correlation with the HRV trends. We removed occupation, which is correlated with education level; planned pregnancy; and marital status, as there were few samples of unplanned pregnancy and not married or cohabitation marital status.

We included data from all the participants in the second trimester and postpartum period analyses. However, we removed the data of 12% (7/58) of the participants from the third trimester analysis owing to preterm births. It should be noted that 43% (25/58) of the participants had term delivery before the 40th gestational week and 21% (12/58N) of the participants had delivery after the 40th gestational week.

We also used HLM models to compare the second trimester with the third trimester, the second trimester with the postpartum period, and the third trimester with the postpartum period. For these analyses, we used HRV parameters as the dependent variable, time (days) as a within-person independent variable, and 1 binary independent between-person variable showing the comparing periods. We also included age, BMI, and education level in the analysis. The HLM model enabled us to perform this multilevel statistical analysis, comparing HRV trends between 2 time frames. Similarly, we included only the participants with term birth in the third trimester. All the analyses were performed using the statsmodels library in Python [38].

### Ethics Approval

This study received ethics approval from the Ethics Committee of the Hospital District of Southwest Finland (Dnro: 1/1801/2018). Written informed consent was obtained from all the participants.

## Results

### Overview

In this section, we present the HR and HRV parameters collected during the second and third trimesters and the 3-month postpartum period. We also present the correlation between HR and HRV trends and age, BMI, and education level. Then, we compare the trends of HRV parameters between the second and third trimesters and between each trimester and the 3-month postpartum period. Data from 58 women were included in this study. The results include 166.5 (SD 46.9) reliable night data per participant during the study, with a total of 9826 night data (70% of possible data) included in this study.

### Second Trimester

A total of 77.70% (4123/5306) of reliable night data were collected in the second trimester. On average, each participant had 69.9 (SD 15.1) reliable night data in the second trimester. HLM model results showed that HR increased significantly, whereas the time-domain parameters (AVNN, SDNN, nSDNN, RMSSD, and nRMSSD) and the frequency-domain parameters (LF, HF, and nHF) decreased significantly during the second trimester. In addition, the results showed no significant association of BMI and education level with HR and HRV trends in the second trimester. However, there was a significant association between age and nSDNN, nRMSSD, HF, and LF/HF. Increase in age was associated with a slight decrease in nSDNN, nRMSSD, and HF and a slight increase in LF/HF. Tables 3 and 4 show the intercept; slope of changes; association of age, BMI, and education level with trends; and the average HR and HRV parameters at the end of the second trimester.

**Table 3:** HR^a^ and time-domain HR variability trends during the second and third trimesters and the postpartum period.

Periods and variables			HR		AVNN^b^		SDNN^c^		nSDNN^d^		RMSSD^e^		nRMSSD^f^
**Second trimester**													
	Intercept (*P* value)	62.736 (<.001)		916.443 (<.001)		84.023 (<.001)		9.079 (<.001)		89.293 (<.001)		9.986 (<.001)	
	Slope (*P* value)	0.045 (<.001)		–0.585 (<.001)		–0.082 (<.001)		–0.006 (<.001)		–0.103 (<.001)		–0.007 (<.001)	
	Age, coefficient (*P* value)	0 (.99)		0.748 (.77)		–0.737 (.09)		–0.080 (.047)		–0.774 (.07)		–0.091 (.03)	
	BMI, coefficient (*P* value)	0.141 (.19)		–1.211 (.38)		–0.059 (.80)		–0.002 (.93)		–0.001 (.99)		0.002 (.93)	
	Education level, coefficient (*P* value)	–0.816 (.46)		14.151 (.32)		1.801 (.47)		0.112 (.64)		2.440 (.29)		0.189 (.41)	
	Final values, mean (SD)	71.1 (7.08)		853 (85)		53.2 (18.05)		6.2 (1.8)		58.7 (22.1)		6.7 (2.1)	
**Third trimester**													
	Intercept (*P* value)	81.324 (<.001)		708.181 (<.001)		69.787 (<.001)		9.237 (<.001)		70.275 (<.001)		8.366 (<.001)	
	Slope (*P* value)	–0.025 (.006)		0.345 (.002)		0.084 (<.001)		0.007 (<.001)		0.071 (<.001)		0.006 (.97)	
	Age, coefficient (*P* value)	–0.131 (.55)		1.905 (.45)		–1.063 (.02)		–0.135 (.002)		–0.984 (.009)		–0.092 (.20)	
	BMI, coefficient (*P* value)	0 (.97)		0.278 (.83)		–0.049 (.83)		–0.009 (.68)		–0.003 (.99)		–0.021 (.57)	
	Education level, coefficient (*P* value)	–1.540 (.19)		17.459 (.20)		5.678 (.01)		0.462 (.049)		6.755 (.001)		1.044 (.006)	
	Final values, mean (SD)	68.6 (7.2)		886.9 (99.5)		65.8 (24.7)		7.2 (2.8)		65.5 (26.6)		7.5 (3.1)	
**Postpartum period**													
	Intercept (*P* value)	47.237 (<.001)		1216.255 (<.001)		115.321 (<.001)		9.481 (<.001)		135.233 (<.001)		11.620 (<.001)	
	Slope (*P* value)	–0.009 (.37)		0.130 (.46)		0.001 (.95)		–0.001 (.69)		–0.037 (.12)		–0.004 (.01)	
	Age, coefficient (*P* value)	0.289 (.099)		–4.729 (.14)		–1.320 (.009)		–0.086 (.03)		–1.857 (.001)		–0.139 (.002)	
	BMI, coefficient (*P* value)	0.097 (.33)		–1.258 (.48)		–0.161 (.56)		–0.003 (.90)		–0.154 (.62)		–0.007 (.76)	
	Education level, coefficient (*P* value)	–0.300 (.77)		5.721 (.76)		–0.122 (.97)		–0.053 (.82)		1.168 (.71)		0.061 (.81)	
	Final values, mean (SD)	58.5 (5.9)		1037.7 (105.3)		67.3 (21.6)		6.5 (2)		69 (24.2)		6.7 (2.2)	

^a^HR: heart rate.

^b^AVNN: average normal interbeat intervals.

^c^SDNN: SD of normal interbeat intervals.

^d^nSDNN: normalized SDNN.

^e^RMSSD: root mean square of the successive difference of normal interbeat intervals.

^f^nRMSSD: normalized RMSSD.

**Table 4:** Trends of frequency-domain heart rate variability parameters during the second and third trimesters and the postpartum period.

Periods and variables			LF^a^		nLF^b^		HF^c^		nHF^d^		LF/HF		nLF/nHF
**Second trimester**													
	Intercept (*P* value)	1045.893 (<.001)		2.422 (<.001)		2990.343 (<.001)		4.727 (<.001)		–0.248 (<.001)		0.565 (.002)	
	Slope (*P* value)	–3.109 (<.001)		0.001 (.72)		–4.224 (<.001)		–0.007 (.003)		–0.061 (.10)		0.001 (.007)	
	Age, coefficient (*P* value)	–6.285 (.62)		–0.031 (.17)		–44.543 (.045)		–0.046 (.32)		2.775 (.001)		–0.003 (.57)	
	BMI, coefficient (*P* value)	2.167 (.75)		0.012 (.33)		–9.352 (.42)		0.035 (.17)		0.766 (.12)		0.002 (.48)	
	Education level, coefficient (*P* value)	48.903 (.50)		0.173 (.17)		71.438 (.55)		0.017 (.95)		–3.943 (.43)		0.033 (.23)	
	Final values, mean (SD)	677.7 (517.4)		2.3 (1.4)		1085.2 (1220.7)		3.7 (1.9)		0.83 (0.55)		0.8 (0.4)	
**Third trimester**													
	Intercept (*P* value)	1008.979 (.03)		4.248 (.001)		1743.362 (.01)		6.166 (.001)		0.67 (.84)		0.995 (.003)	
	Slope (*P* value)	0.424 (.44)		0.006 (.09)		2.767 (.007)		0.004 (.21)		–0.097 (.11)		0.001 (.28)	
	Age, coefficient (*P* value)	–19.456 (.12)		–0.070 (.03)		–40.218 (.04)		–0.098 (.054)		2.440 (.008)		–0.004 (.68)	
	BMI, coefficient (*P* value)	1.655 (.80)		–0.018 (.28)		–5.994 (.53)		–0.001 (.97)		0.367 (.44)		–0.006 (.17)	
	Education level, coefficient (*P* value)	92.269 (.17)		0.238 (.17)		221.413 (.02)		0.261 (.35)		–5.113 (.28)		0.030 (.51)	
	Final values, mean (SD)	926.9 (956.7)		2.6 (1.9)		1607.7 (2156.1)		4.4 (2.5)		0.77 (0.40)		0.7 (0.4)	
**Postpartum period**													
	Intercept (*P* value)	2124.653 (.002)		2.486 (.09)		4969.652 (<.001)		6.670 (.001)		–0.397 (.27)		0.416 (.02)	
	Slope (*P* value)	2.415 (.05)		–0.005 (.40)		–0.682 (.63)		–0.001 (.77)		0.234 (.004)		–0.002 (.05)	
	Age, coefficient (*P* value)	–29.427 (.14)		–0.012 (.78)		–94.705 (<.001)		–0.054 (.33)		3.498 (<.001)		0.006 (.25)	
	BMI, coefficient (*P* value)	0.327 (.98)		0.023 (.33)		–13.985 (.32)		0.022 (.47)		0.528 (.35)		0.002 (.40)	
	Education level, coefficient (*P* value)	–50.704 (.64)		0.279 (.24)		22.307 (.88)		–0.291 (.36)		–5.332 (.35)		0.053 (.06)	
	Final values, mean (SD)	1307.9 (920.6)		2.5 (2.5)		1486.4 (1327.6)		5.3 (2.8)		1 (0.48)		0.6 (0.3)	

^a^LF: low-frequency power.

^b^nLF: normalized LF.

^c^HF: high-frequency power.

^d^nHF: normalized HF.

### Third Trimester

During the third trimester, 70.25% (2716/3866) of reliable night PPG data were collected. Each participant had an average of 53.2 (SD 15.1) reliable night PPG data in the third trimester. The HLM models show that HR decreased significantly, whereas the time-domain parameters (AVNN, SDNN, nSDNN, and RMSSD) and frequency-domain parameter (HF) increased significantly during the third trimester (refer to Tables 3 and 4 for details). Moreover, the results indicated that high education level was associated with high SDNN, nSDNN, RMSSD, nRMSSD, and HF. It also showed that increase in age was associated with a slight decrease in SDNN, nSDNN, RMSSD, nLF, and HF and a slight increase in LF/HF.

Considering both trimesters as a whole, the models indicated that HR significantly increased (*P*<.001), whereas AVNN, SDNN, RMSSD, LF, and HF decreased during pregnancy (with *P*<.001, *P*=.04, *P*=.001, *P*=.44, *P*=.44, respectively). However, during the last weeks of pregnancy, starting from pregnancy week 35, HR began to decrease and HRV parameters (AVNN, SDNN, RMSSD, LF, and HF) began to increase, but they did not reach the level of pregnancy week 16 before the delivery.

### Postpartum Period

**Figure 2:**
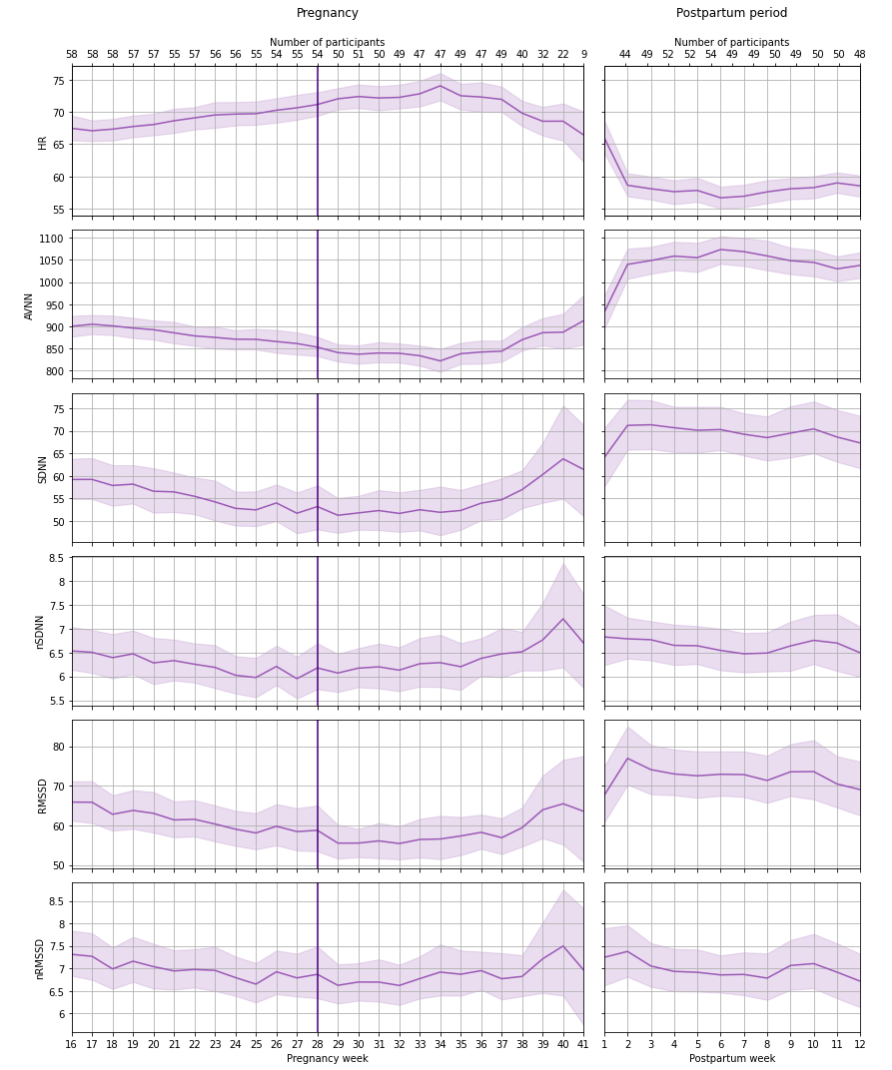
Weekly mean and 95% CI of heart rate (HR) and time-domain HR variability parameters during pregnancy and postpartum period. The number of participants with reliable data per week is also indicated. The vertical line indicates pregnancy week 28 and separates the second and third trimesters. AVNN: average normal interbeat intervals; RMSSD: root mean square of the successive differences of normal interbeat intervals; nRMSSD: normalized RMSSD; SDNN: SD of normal interbeat intervals. nSDNN: normalized SDNN.

**Figure 3:**
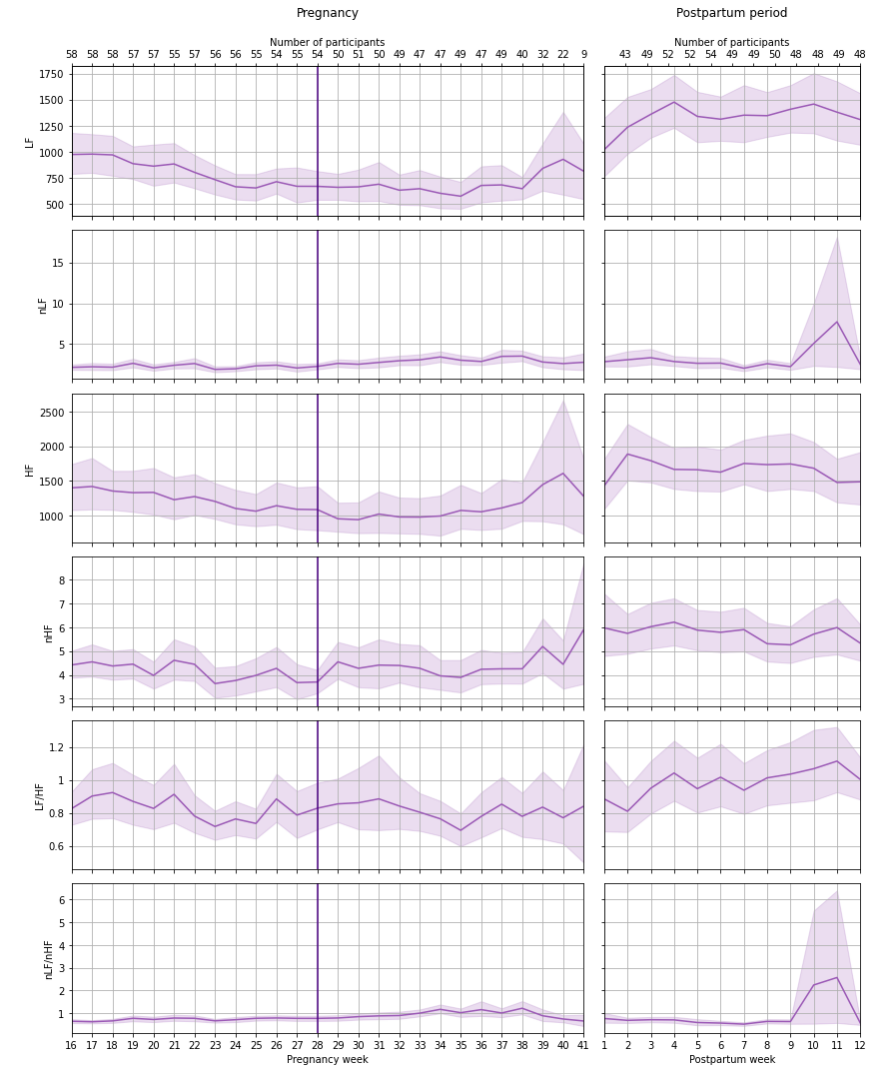
Weekly mean and 95% CI of frequency-domain heart rate variability parameter during pregnancy and postpartum period. The number of participants with reliable data per week is also indicated. The vertical line indicates pregnancy week 28 and separates the second and third trimesters. HF: high-frequency power; LF: low-frequency power; nHF: normalized HF; nLF: normalized LF.

In the postpartum period, 62.05% (2987/4814) of reliable night PPG data were collected from the participants. Each participant had an average of 53.4 (SD 19.7) reliable night data in this period. During the first 12 weeks after delivery, the time-domain parameter (nRMSSD) decreased slightly and the frequency-domain parameter (LF/HF) increased slightly (Tables 3 and 4). The results indicated that increase in age was associated with a slight decrease in SDNN, nSDNN, RMSSD, nRMSSD, and HF and a slight increase in LF/HF. Moreover, the results showed no significant correlation between the duration of pregnancy and trends of HR and HRV parameters during postpartum period. Figures 2 and 3 represent the trends of HR, time-domain, and frequency-domain HRV parameters during pregnancy and the 3-month postpartum period.

### Comparison of Trends in HRV Parameters Among the Second Trimester, Third Trimester, and 3-Month Postpartum Period

We compared the trends of HRV parameters among the second trimester, third trimester, and postpartum period.

#### The Second and Third Trimesters

The time-domain HRV parameters, including AVNN, SDNN, nSDNN, and RMSSD, and some frequency-domain parameters, including LF, HF, and nHF, were slightly higher in the third trimester than in the second trimester. Moreover, HR was slightly high in the second trimester.

The average increase in HR from the beginning of the second trimester to week 34, when HR reached its highest level was 6.58 beats per minute (bpm). Time-domain parameters, AVNN, SDNN, nSDNN, RMSSD, and nRMSSD, were 77.3, 7.8, 0.4, 9.5, 0.5 milliseconds lower, respectively, at the beginning of the third trimester than the beginning of the second trimester. In addition, the slope of per-day changes in HR was slightly lower, whereas this slope was slightly higher for AVNN, SDNN, nSDNN, RMSSD, nRMSSD, LF, HF, and nHF in the third trimester than in the second trimester (Tables 5 and 6). Moreover, the decrease in HRV trends, including SDNN, nSDNN, RMSSD, nRMSSD, and HF, in the third trimester compared with the second trimester was slightly higher in younger women than in older women. However, in this comparison, the difference in LF/HF slightly increased with increase in age.

**Table 5:** Comparison of HR^a^ and time-domain HR variability parameters between the second and third trimesters, second trimester and postpartum period, and third trimester and postpartum period.

Comparisons and variables.			HR		AVNN^b^		SDNN^c^		nSDNN^d^		RMSSD^e^		nRMSSD^f^
**Second trimester and third trimester**													
	Slope (*P* value)	−0.080 (<.001)		1.052 (<.001)		0.149 (<.001)		0.010 (<.001)		0.168 (<.001)		0.011 (<.001)	
	Intercept (*P* value)	6.128 (<.001)		−77.279 (<.001)		−7.793 (<.001)		−0.377 (<.001)		−9.535 (<.001)		−0.529 (<.001)	
	Age (years), coefficient (*P* value)	0.015 (.93)		0.017 (.99)		−0.859 (.03)		−0.090 (.02)		−1.097 (.006)		−0.119 (.003)	
	BMI (kg/m^2^), coefficient (*P* value)	0.125 (.23)		−1.316 (.32)		−0.101 (.65)		−0.007 (.75)		−0.071 (.74)		−0.002 (.94)	
	Education level, coefficient (*P* value)	−1.396 (.20)		19.196 (.16)		2.257 (.33)		0.153 (.50)		3.689 (.10)		0.274 (.23)	
**Second trimester and postpartum period**													
	Slope (*P* value)	−0.061 (<.001)		0.800 (<.001)		0.079 (<.001)		0.004 (.009)		0.077 (<.001)		0.003 (.02)	
	Intercept (*P* value)	−8.135 (<.001)		133.327 (<.001)		10.458 (<.001)		0.235 (.002)		7.776 (<.001)		−0.062 (.32)	
	Age (years), coefficient (*P* value)	0.071 (.68)		−1.067 (.68)		−0.843 (.046)		−0.086 (.02)		−1.208 (.009)		−0.114 (.005)	
	BMI (kg/m^2^), coefficient (*P* value)	0.122 (.22)		−1.202 (.41)		−0.047 (.84)		0.005 (.83)		−0.046 (.86)		0.002 (.94)	
	Education level, coefficient (*P* value)	−0.780 (.44)		11.211 (.45)		1.493 (.55)		−0.005 (.98)		2.057 (.42)		0.138 (.55)	
**Third trimester and postpartum period**													
	Slope (*P* value)	0.026 (<.001)		−0.306 (.003)		−0.101 (<.001)		−0.008 (<.001)		−0.102 (<.001)		−0.009 (<.001)	
	Intercept (*P* value)	−14.686 (<.001)		212.495 (<.001)		19.492 (<.001)		0.691 (<.001)		18.524 (<.001)		0.541 (<.001)	
	Age (years), coefficient (*P* value)	0.121 (.47)		−1.801 (.47)		−0.921 (.02)		−0.080 (.03)		−1.142 (.002)		−0.108 (.003)	
	BMI (kg/m^2^), coefficient (*P* value)	0.097 (.29)		−1.538 (.27)		−0.132 (.55)		0.001 (.98)		−0.193 (.36)		−0.010 (.64)	
	Education level, coefficient (*P* value)	−1.330 (.16)		15.663 (.28)		2.892 (.21)		0.243 (.25)		4.053 (.06)		0.381 (.08)	

^a^HR: heart rate.

^b^AVNN: average normal interbeat intervals.

^c^SDNN: SD of normal interbeat intervals.

^d^nSDNN: normalized SDNN.

^e^RMSSD: root mean square of the successive difference of normal interbeat intervals.

^f^nRMSSD: normalized RMSSD.

**Table 6:** Comparison of frequency-domain heart rate variability parameters between the second and third trimesters, second trimester and postpartum period, and third trimester and postpartum period.

Comparisons and variables			LF^a^		nLF^b^		HF^c^		nHF^d^		LF/HF		nLF/nHF
**Second trimester** **and** **third trimester**													
	Slope (*P* value)	2.776 (<.001)		0.003 (.29)		5.628 (<.001)		0.010 (.002)		−0.061 (.25)		0 (.70)	
	Intercept (*P* value)	−248.629 (<.001)		0.396 (<.001)		−371.246 (<.001)		−0.419 (.005)		0.338 (.89)		0.172 (<.001)	
	Age (years), coefficient (*P* value)	−12.053 (.29)		−0.046 (.06)		−58.317 (.004)		−0.063 (.13)		0.027 (.003)		−0.003 (.61)	
	BMI (kg/m^2^), coefficient (*P* value)	−0.220 (.97)		0.003 (.82)		−10.722 (.34)		0.023 (.34)		0.005 (.30)		0 (.93)	
	Education level, coefficient (*P* value)	42.367 (.52)		0.171 (.22)		105.380 (.36)		0.051 (.84)		−0.041 (.43)		0.023 (.46)	
**Second trimester** **and** **postpartum period**													
	Slope (*P* value)	5.261 (<.001)		−0.007 (.045)		3.748 (<.001)		0.002 (.57)		0.287 (<.001)		−0.003 (<.001)	
	Intercept (*P* value)	194.724 (<.001)		0.754 (<.001)		311.128 (<.001)		1.073 (<.001)		−1.367 (.63)		0.061 (.16)	
	Age (years), coefficient (*P* value)	−12.415 (.36)		−0.025 (.36)		−64.949 (.004)		−0.048 (.28)		3.152 (.001)		0.001 (.85)	
	BMI (kg/m^2^), coefficient (*P* value)	1.922 (.80)		0.028 (.08)		−8.505 (.49)		0.030 (.23)		0.706 (.16)		0.003 (.27)	
	Education level, coefficient (*P* value)	36.128 (.65)		0.223 (.16)		69.474 (.58)		−0.013 (.96)		−3.490 (.48)		0.066 (.01)	
**Third trimester and postpartum period**													
	Slope (*P* value)	1.773 (.05)		−0.015 (.002)		−3.219 (.001)		−0.007 (.12)		0.377 (<.001)		−0.005 (<.001)	
	Intercept (*P* value)	496.288 (<.001)		0.240 (.28)		765.852 (<.001)		1.488 (<.001)		−2.648 (.40)		−0.160 (.008)	
	Age (years), coefficient (*P* value)	−17.497 (.22)		−0.039 (.23)		−65.002 (.001)		−0.079 (.09)		2.629 (.004)		0.002 (.79)	
	BMI (kg/m^2^), coefficient (*P* value)	2.643 (.73)		0.011 (.56)		−13.823 (.20)		0.012 (.65)		0.530 (.32)		−0.002 (.63)	
	Education level, coefficient (*P* value)	18.376 (.82)		0.224 (.23)		142.030 (.19)		0.028 (.92)		−5.352 (.30)		0.045 (.28)	

^a^LF: low-frequency power.

^b^nLF: normalized LF.

^c^HF: high-frequency power.

^d^nHF: normalized HF.

#### The Second Trimester and Postpartum Period

In the postpartum period, HR was significantly lower (on average 8.1 bpm), and the time-domain parameters, AVNN (133.3 milliseconds), SDNN (10.5 milliseconds), nSDNN (0.2 milliseconds), RMSSD (7.8 milliseconds), and frequency-domain parameters LF (195.1 square milliseconds), nLF (0.7 square milliseconds), and HF (312.4 square milliseconds) were significantly higher than those in the second trimester. The slope of changes in HR was slightly lower, whereas the slope of changes in AVNN, SDNN, nSDNN, RMSSD, nRMSSD, LF, nLF, HF, LF/HF, and nLF/nHF was higher than those in the second trimester (Tables 5 and 6). The difference between the trends of SDNN, nSDNN, RMSSD, nRMSSD, and HF decreased, whereas the difference in LF/HF slightly increased with increase in age.

#### The Third Trimester and Postpartum Period

In the postpartum period, HR was significantly lower (on average 14.7 bpm) than that at the beginning of the third trimester. However, the time-domain parameters, AVNN, SDNN, nSDNN, RMSSD, and nRMSSD, and frequency-domain parameters, LF, HF, and nHF were, on average, 212.8 milliseconds, 19.4 milliseconds, 0.7 milliseconds, 18.5 milliseconds, 0.5 milliseconds, 495 square milliseconds, 764.6 square milliseconds, and 1.5 square milliseconds higher, respectively, at the beginning of the postpartum period than at the beginning of the third trimester. The slope of changes in AVNN, SDNN, nSDNN, RMSSD, nRMSSD, nLF, HF, and nHF was slightly higher, whereas the slope of changes in LF was slightly lower in the third trimester than in the postpartum period (Tables 5 and 6). In addition, the difference between the trends of SDNN, nSDNN, RMSSD, nRMSSD, and HF decreased, whereas the difference in LF/HF slightly increased with increase in age.

## Discussion

### Principal Findings

Our results show that HR increased significantly during the second trimester, whereas it slightly decreased during the third trimester. During the postpartum period, HR continued to decrease, but the reduction was not statistically significant; however, compared with pregnancy, HR was significantly low during the postpartum period. On average, HR increased by 6.6 bpm from 16 weeks to 34 weeks of gestation, after which, it started to decrease.

The trends detected in this study are consistent with the previous review by Loerup et al [2], in which the mean increase was 7.6 (95% CI 1.8-13.4) bpm from 10 weeks to 40 weeks of gestation. The increase in HR during pregnancy is considered physiological and explained by elevated blood volume, which results in increased cardiac output [7].

Regarding HRV, the time-domain parameters and their normalized values decreased significantly during the second trimester and, then, increased significantly during the third trimester. However, these parameters did not reach the level of those during the second trimester. In the postpartum period, the time-domain parameters were stable, and only nRMSSD decreased. Regarding the frequency-domain parameters, LF, HF, and nHF decreased significantly during the second trimester, whereas nLF/nHF increased slightly. During the third trimester and postpartum period, the parameters were stable, except HF, which increased, and LF/HF, which decreased slightly. Changes in HRV parameters occur owing to the pregnancy and the physiological changes in the woman’s body [1]. The trend in HRV parameters during pregnancy was decreasing, with values returning to normal after delivery [3-5].

The results indicated that BMI is not significantly associated with HRV trends. In addition, younger women had higher nSDNN, nRMSSD, and HF in the second trimester and lower SDNN, nSDNN, RMSSD, nRMSSD, and HF and slightly lower LF/HF during the third trimester and postpartum period than older women. These results are consistent with previous studies showing a negative correlation between age and HRV parameters [25]. Furthermore, the results showed that more educated women had higher SDNN, nSDNN, RMSSD, nRMSSD, and HF during the third trimester than the less educated women, which may indicate low stress level in highly educated women. Previous studies also showed that highly educated people experienced low stress in a stressful situation [39], and low education level is identified as a determinant of stress during pregnancy [40].

### Comparison With Previous Work

To the best of our knowledge, this is the first paper that describes and evaluates HR and HRV parameters measured using PPG signals continuously during pregnancy and the postpartum period in participants’ normal daily lives during the night. Previous studies have been limited to a few samples assessed in controlled environments during pregnancy and the postpartum period. Only Stein et al [1] performed a study in free-living conditions; they measured HRV using Holter ECG for 24-hour periods during pregnancy.

Continuously measured HR followed the physiological trends, increasing as the pregnancy proceeded and returning to normal during the postpartum period. However, it is notable that in this study, we did not measure HR levels before pregnancy; thus, it is not possible to confirm whether HR levels returned to prepregnancy levels during the 3-month follow-up. Several studies have shown that HR increases during pregnancy [7,15,21,24] and decreases again during the postpartum period [4]. In this study, the detected increase during pregnancy followed the results of the meta-analysis, which included >10,000 HR measurements from >8000 women [2]. The small difference may be explained by the small sample size in this study and differences in the measurement periods, as our study measurements started at gestational week 16 and the meta-analysis started from gestational week 10 [2]. However, it is suggested that most of the changes in cardiac autonomic modulation occur within the first weeks after conception [1]; thus, in this study, we were not able to detect the early changes.

Interestingly, according to our study, HR was the highest during pregnancy week 34 and started to decrease thereafter. In many previous studies [1,23,24], the last measurement points were before week 36; thus, the decrease in HR at the end of the pregnancy may not have been captured. On the basis of the meta-analysis by Loerup et al [2], a few previous studies have shown a slight reduction in HR at the end of pregnancy. However, most studies show a continuous increase during pregnancy.

The results showed that all the time-domain HRV parameters measured in this study and the frequency-domain parameters (LF and HF) decreased during the second trimester. Furthermore, most of the measured parameters (AVNN, SDNN, RMSSD, LF, and HF) showed decreasing trends throughout pregnancy. On the basis of previous studies with intermittent measurements, the trends of different HRV parameters decreased during the course of pregnancy [1,7,9,11,15,22-24]. Some studies also found increasing trends, for example, in LF [23], and some did not find any significant changes during pregnancy [16,21]. These conflicting results are partly owing to different methodological choices, such as limited HRV recordings and a small number of participants, but they also reflect the challenges of measuring and interpreting HRV [25].

On the basis of the continuous measurements in our study, a change from a decreasing trend to a slightly increasing trend in HRV parameters was observed during the last weeks of pregnancy, starting at week 35. Most previous studies included very few or no measurements of HRV after pregnancy week 35; therefore, the change has probably not been detected [1,9,22-24]. Long intervals (eg, weeks) between successive HRV measurements restrict the findings regarding fine-grained trends at the end of pregnancy. Continuous measurements provide opportunities to detect small changes also. In Finland, pregnant women are entitled to maternity leave starting 5 to 8 weeks before the estimated delivery date [41]. Thus, we could speculate that one explanation for the changes in HR and HRV parameters around gestational weeks 34 to 35 could be the beginning of the maternity leave. Maternity leave allows, for example, a woman to modify her daily rhythm, and therefore, the level of stress may decrease. However, this issue requires more research in the future. It is also notable that the data for this study were collected partly during the COVID-19 pandemic and the first wave of restrictions, which may have affected the behavior and, by implication, changes in the physiological parameters of the participating women [42].

Some HRV parameters were negatively associated with age, whereas LF/HF was positively associated with age, in our study. Several studies have shown similar correlations between age and HRV parameters [25,43]. Changes in HRV parameters are also associated with stress, as HRV represents the ability of the heart to respond to a variety of psychological and environmental stimuli [44]. Although cardiovascular changes during pregnancy are physiological, Klinkenberg et al [13] suggested that psychosocial stress also affects HRV parameters in pregnant women. Low values of SDNN, RMSSD, and HF and high values of LF and LF/HF may indicate mental stress [14]. Our results showed a positive correlation between HRV parameters and education level in the third trimester, which may indicate low stress level in highly educated women [39,40]. However, interpreting HRV parameters regarding stress is difficult owing to physiological changes during pregnancy and the variety of potential stressors and individual stress responses [25,44].

During the postpartum period, some of the HRV parameters (SDNN, RMSSD, LF, and HF) increased as expected, as the body recovers from the pregnancy and delivery and returns to the *normal* nonpregnant state [1,5,19]. It is suggested that autonomic nervous system recovers approximately 4 months after delivery [3]. On the basis of only one HRV measurement during the third trimester and another at 3-month postpartum period, Heiskanen et al [4] found similar results regarding the frequency-domain HRV parameters; the parameters significantly increased from the third trimester to the postpartum period. They suggest that the optimal time for measuring the recovery of HRV is 6 months after childbirth; however, possible new pregnancy or the use of oral contraceptives may affect the results at that point [4]. In a recently published study by Brown et al [5], the only significant change in HRV was observed between the third trimester and 4 to 6 weeks of the postpartum period.

In this study, we continuously collected HRV data from pregnant women using an IoT-based maternal monitoring system [26]. The system could collect a considerable amount of HRV data. We were able to extract reliable data from >70% of possible nights during pregnancy and >60% of nights after delivery. The results indicate that continuous HRV monitoring with PPG signals can be used in free-living conditions during pregnancy and the postpartum period. In contrast to previous studies, our results contained fine-grained HRV data, which enabled us to investigate the trends with more granularity.

HRV monitoring during pregnancy could be used for the early detection of complications, such as gestational hypertension [6] and pre-eclampsia [8,9], as reflected in previous studies. For example, Hossen et al [8] developed a model based on frequency-domain HRV parameters to distinguish between pre-eclampsia and normal pregnancy. In addition to interesting HRV trends during pregnancy and the postpartum period, the results of this study showed the feasibility of the IoT-based system for remote HRV monitoring of maternal health. This system can be further developed to build a personalized model that uses individual parameters and normal HRV trends for early anomaly detection. This model may even provide early warning for mothers in a noninvasive and cost-efficient manner. This technology could provide a solution to support maternal health services in low- and middle-income countries. Although many other efforts are also needed [45], technology could enhance health equality between urban and rural areas.

### Limitations

Women with both high-risk and low-risk pregnancies were included in the sample; however, no differences were detected in HRV parameters between the 2 groups, and therefore, the sample was considered as one group. Only nighttime data were used for the analyses; the minimum resting HR between midnight and 6 AM was used to analyze the trend of HR and corresponding HRV parameters to minimize the effect of noises and artifacts [25]. HRV was measured using PPG signals, which were collected with a frequency of 20 Hz. Therefore, our results need to be interpreted with caution, as not all HRV parameters can be obtained reliably at this frequency [26,35]. Moreover, some studies suggest that HRV changes occur mostly during early pregnancy [1,19], and these changes could not be detected in this study because data collection started at pregnancy week 16. Our future work will consider using high-frequency PPG signals to study other HRV trends. Furthermore, when the participants are involved in different activities, daytime HRV parameters would also provide interesting data if the noises and artifacts caused by movement could be removed from the data. In addition, it would be important to control the HRV analysis for various confounding factors such as medical conditions (eg, hypertension) and mental distress.

### Conclusions

In this study, we conducted continuous long-term measurements of HR and HRV from pregnancy week 16 to 3 months of the postpartum period during participants’ daily lives. The measurements were performed through the collection of PPG signals from wearable smartwatches. The results showed that HR and HRV mainly followed the expected and previously reported trends; HR increased and HRV parameters decreased as pregnancy proceeded, and the values returned to normal after delivery. These trends reflect the normal physiological changes during pregnancy and postpartum period. However, from the continuous measurements, we detected that HR started to decrease and HRV parameters started to increase during the last weeks of pregnancy. This issue needs more research in the future. The results also showed a positive association between HRV parameters and education level in the third trimester. Furthermore, our results showed that using PPG signals, it is possible to follow HRV continuously in free-living conditions. Our system could be further developed and used in the future; for example, to detect abnormalities during pregnancy.

## Abbreviations

AVNN: average normal interbeat intervals
bpm: beats per minute
ECG: electrocardiogram
HF: high-frequency power
HLM: hierarchical linear mixed
HR: heart rate
HRV: heart rate variability
IBI: interbeat interval
IoT: Internet of Things
LF: low-frequency power
nHF: normalized high-frequency power
nLF: normalized low-frequency power
nRMSSD: normalized root mean square of the successive difference of normal interbeat intervals
nSDNN: normalized SD of normal interbeat intervals
PPG: photoplethysmogram
RMSSD: root mean square of the successive difference of normal interbeat intervals
SDNN: SD of normal interbeat intervals
